# Common Pitfalls in Ewing Sarcoma and Desmoplastic Small Round Cell Tumor Diagnosis Seen in a Study of 115 Cases

**DOI:** 10.3390/medsci9040062

**Published:** 2021-10-15

**Authors:** Nikolaos A. Trikalinos, John S. A. Chrisinger, Brian A. Van Tine

**Affiliations:** 1 Division of Medical Oncology, Washington University in St. Louis, St. Louis, MO 63110, USA; bvantine@wustl.edu; 2 Siteman Cancer Center, St. Louis, MO 63110, USA; 3 Department of Pathology and Immunology, Washington University in St. Louis, St. Louis, MO 63110, USA; jschrisi@wustl.edu; 4 Department of Pediatric Hematology/Oncology, St. Louis Children’s Hospital, St. Louis, MO 63110, USA

**Keywords:** sarcoma, Ewing, desmoplastic small round cell tumor, diagnostic errors, neuroendocrine tumors

## Abstract

Ewing sarcoma (ES), “Ewing-like sarcoma” (ELS) and desmoplastic small round cell tumors (DSRCT) can masquerade as other tumor types, particularly neuroendocrine neoplasms and receive inappropriate treatment. We retrieved 115 cases of ES, ELS and DSRCT seen over 17 years in a tertiary center. An initial misdiagnosis or incomplete diagnosis occurred in 6/93 (6.4%) of ES/ELS and 5/22 (22.7%) of DSRCT cases. The most frequent misdiagnosis was small cell neuroendocrine carcinoma. While any misdiagnosis or incomplete classification is almost certainly multifactorial, the most common identified reason for erroneous/incomplete initial reporting was expression of neuroendocrine markers. Other contributing factors included keratin expression, older patient age and apparently unusual tumor location. Most patients treated with a non-sarcoma chemotherapy regimen expired, while those who received a sarcoma-related regimen were alive as of last evaluation. Increased awareness of this diagnostic pitfall is needed in evaluating cases of round cell malignancies.

## 1. Introduction

Ewing sarcoma (ES) and desmoplastic small round cell tumors (DSRCT) are aggressive, rare sarcomas with a respective incidence of about 1 case and 0.5 cases per million [1] per year in North America. Both commonly present with a round cell pattern, but they can be differentiated by a combination of clinical, histologic, immunohistochemical and molecular findings. This includes the presence of EWSR1-FLI1, or much less commonly EWSR1-ERG fusions in 95% of ES and EWSR1-WT1 fusion in DSRCT [2]. Other undifferentiated small round cell sarcomas (USRCS), some previously known as “Ewing-like sarcomas” (ELS), make up a diverse group of sarcomas with round (and not so round) cytomorphology including round cell sarcomas with EWSR1-non-ETS fusions, CIC-rearranged sarcoma and sarcoma with BCOR genetic alterations [3].

Despite their relative aggressiveness, rapid identification and initiation of long-term chemotherapy, in combination with surgical and radiation approaches, can have substantial effect on the outcomes of these diseases. For example, five-year survivals can reach up to 70% for localized [4] and up to 20% of metastatic ES patients [5]; DSCRTs are generally responsive to systemic treatments and neoadjuvant chemoradiotherapy followed by surgery can lead to up to 55% 3-year overall survival [6,7]. The course of USRCS varies; CIC-rearranged sarcomas exhibit highly aggressive behavior [8], while the outcome of BCOR-CCNB3 sarcoma is similar to ES [9,10]. Unfortunately, distinguishing ES, ELS and DSRCT from other neoplasms can be challenging, risking misdiagnosis. This can lead to suboptimal outcomes and missed opportunities for cure.

In our institutional experience, we have on occasion seen patients with ES, ELS and DSCRT whose initial diagnosis was erroneous/incomplete; in some the misdiagnosis was almost immediately detected, whereas in others, it was uncovered only after a variety of non-sarcoma treatments. We thus sought to estimate the rate of this reclassification and understand the reasons behind it and potential implications for patient outcomes.

## 2. Materials and Methods

Under an institutional review board (IRB) approval, we used a coding algorithm to retrieve pathology files of patients diagnosed with ES, ELS (term previously used for a subset of sarcomas which by modern diagnostic methods would likely be classified as USRCS of various types) and DSCRT from 2003 to 2020 at our institution. We documented the initial diagnosis from the pathology report or clinical consultation note (including copies of outside records provided for routine patient care), and whether it differed from our final classification. We documented the exact pathognomonic molecular alterations, when available, as well as the commercial or in-house panel used to detect those. When not available, we relied on note reporting and other indirect knowledge from patient files. When available, representative slides or scanned whole slide images of initially misdiagnosed cases were re-examined by a dedicated sarcoma pathologist. In all initially misclassified or incompletely classified cases, pathology reports were reviewed for reported histologic findings, and immunohistochemical (IHC) and molecular analysis. We extracted basic patient and treatment characteristics and survival status from the electronic medical record. All information was procured from the electronic medical records and pathology departmental archives, no patients or relatives were contacted. 

## 3. Results

Between 2003 and 2020 a total of 131 cases (108 ES/ELS and 23 DSCRT) were identified. For fifteen of these, no usable extra information could be retrieved, limiting our sample to 115 patients (93 ES/ELS and 22 DSCRT). Overall, 11 patients (6 ES/ELS and 5 DSCRT) were initially incompletely classified or misdiagnosed (6.4% for ES/ELS and 22.7% for DSCRT) (Table 1 and Table 2). 

**Figure 1:**
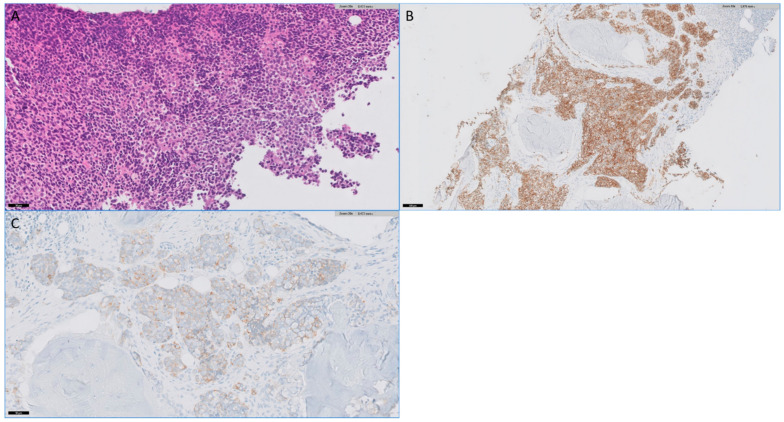
(**A**). Photomicrograph of biopsy from case 4 showing sheet-like growth of monotonous round cells with clear to pale eosinophilic cytoplasm characteristic of Ewing sarcoma (H&E). Prior biopsy was diffusely positive for keratin (**B**) and showed patchy expression of synaptophysin (**C**).

Males comprised 81% of that population which otherwise had a mean age of 40.9 years. In six cases, the initial incomplete or misdiagnosis occurred in tertiary centers (including true pathology consults), while five occurred in community institutions. Of the six cases incompletely or misclassified in tertiary sarcoma centers, three cases were misdiagnosed at our institution, while the remainder originated at outside centers. Seven of the tumors were misdiagnosed as (or favored to be) neuroendocrine carcinoma or carcinoma with neuroendocrine features, while two were labelled as “poorly differentiated or small cell neoplasm”. More rare misdiagnoses included non-Hodgkin lymphoma (1) and high-grade endometrial stromal sarcoma (1). An example with representative photomicrographs is shown in Figure 1.

Median follow-up was 106 days for the entire population, 96 days for correctly diagnosed cases and 152 days for misdiagnosed cases. Treatment details were not available for two patients. Of the rest, 4/9 were ultimately treated with a sarcoma regimen (interval compressed vincristine, doxorubicin, cyclophosphamide alternating with ifosfamide, etoposide VDC/IE), while all others received a platinum regimen (four with carboplatin or cisplatin and one with carboplatin and paclitaxel). Survival status was not known for one patient. Of the remaining ten, 5 were alive and 5 had expired as of last follow-up. Of the alive patients 3/5 (60%) received a sarcoma regimen as first treatment. Of the expired patients, 3/5 (60%) received a non-sarcoma regimen.

Review of the revised diagnosis cases revealed the following patterns. Of cases stained with synaptophysin, chromogranin and/or CD56, expression of neuroendocrine markers was noted in of 5/6 ES/ELS and of 3/4 DSRCT, which appear to have substantially contributed to an initial erroneous impression of a neuroendocrine neoplasm in those five cases of ES/ELS and two cases of DSRCT. Keratin expression was present in 2/6 ES/ELS and characteristic staining with keratin was noted in 4/5 DSRCT. Molecular analysis for EWSR1 rearrangement or relevant EWSR1 fusions was initially undertaken in only 1/6 ES/ELS and 0/5 DSRCT. In the case of the ES initially tested for EWSR1 rearrangement, fluorescence in situ hybridization testing was negative for EWSR1 rearrangement in the first biopsy, while a subsequent biopsy was positive. Of note, this initially EWSR1 rearrangement negative case was positive for keratin (diffuse) and synaptophysin, however it is also noteworthy that this patient was an 11-year-old boy with a femur tumor. Apparent origin in the uterus (involvement of the endometrium and myometrium) in addition to involvement of the adnexa and omentum contributed to a DSRCT being mistaken for a high-grade endometrial stromal sarcoma. Other unusual circumstances contributing to misinterpretation include a include a synaptophysin, chromogranin and CD56 (keratin, desmin and WT1 negative) mesenteric ES (EWSR1-FLI1 positive) and numerous tumor-associated lymphocytes in an ELS originally diagnosed as lymphoma.

## 4. Discussion

In this case series, we examined 115 patients with ES/ELS and DSCRT seen in our tertiary center (both initial presentations and consultations) over more than 15 years, documented all potentially treatment changing incompletely or misdiagnosis cases and speculated on the reasons behind it. Patients came in roughly equal numbers from academia and the community setting. The error rates for DSCRT were significant, about 23%, while for ES/ELS they were close to 6%. Molecular analysis for EWSR1 rearrangement was often very helpful in confirming the diagnosis. We showed that over half of misdiagnosed patients were thought to have, or favored to have, a NEC, about half of them were treated with a non-sarcoma regimen and most of these have expired. Most patients in which a diagnosis was reversed before treatment and who received a sarcoma regimen have remained alive. This is the first study of its kind in an extremely rare population spanning more than 15 years and the first time the mischaracterization process has been studied in such a detailed manner. This has the potential to inform pathologists and clinicians alike of the most common pitfalls in ES/ELS and DSRCT diagnosis.

Our study highlights that identification of ES/ELS and DSRCT can be challenging, and that correct diagnosis is critical for patient outcome. Sarcomas encompass a wide range of more than 100 subtypes [11], and the landscape is rapidly evolving, in large part with the help with improving knowledge of molecular diagnostics. A prospective multi-institutional observational study [12] looked into the effect of molecular profiling in sarcoma subtype misdiagnosis. While all of the patients were appropriately diagnosed with sarcoma on presentation, 53 of 384 patients had their diagnosis changed after inclusion of molecular markers. This included 12% of the Ewing’s sarcoma family of tumors. In our series, molecular analysis was only initially performed in one case, and molecular analysis was eventually diagnostically useful in 9/10 cases in which it was performed. That of course does not mean that testing for EWSR1 rearrangement should be routinely performed in the workup of suggested neuroendocrine neoplasms. However, if the clinical scenario is unusual (e.g., young patient with a bone tumor or young male with apparent peritoneal “carcinomatous”) or the diagnosis is ambiguous (e.g., “small cell neoplasm, favor neuroendocrine carcinoma”) then consultation with the pathologist, and perhaps molecular testing, are warranted. This might become more commonplace with the increased use of broad coverage next-generation sequencing panels. While not an apparent cause of misdiagnosis in our series, cutaneous/subcutaneous ES can easily be mistaken for a primary or metastatic neuroendocrine neoplasm [13]. A note to make is that some of the sarcoma alterations such as BCOR or CIC-DUX4 were not described in the early 2000s. This does not justify the misdiagnosis though; the relevant histologies should still be classified as unclassified sarcomas, even then based on our assessment.

We also believe that in difficult cases, consideration of subspecialist pathologist consultation should be considered at the discretion of the case pathologist. Five of eleven cases in our series were diagnosed at community centers, and of our in-house misdiagnosed cases, none occurred following the full establishment of subspecialization including the staffing of a bone and soft tissue pathology section. That said, the series includes cases misdiagnosed by experts in the field which highlights how difficult correct classification can be in some cases. Poorly differentiated neuroendocrine tumors or small cell were the most common misdiagnoses, even in cases referred from other tertiary centers. A major contributing factor seems to have been the expression of neuroendocrine markers (synaptophysin, chromogranin and/or CD56) with or without co-expression of keratin. A subset of ES express synaptophysin and staining with chromogranin is rarely observed [13,14,15,16,17]. Further expression of CD56 may be present [14,16,17]. INSM1 is a newer marker of neuroendocrine differentiation [18]; however, it is also expressed in approximately 20–30% of ES [19,20,21]. Moreover, keratin expression in sarcomas, including ES/ELS and DSRCT, can contribute to misdiagnosis if interpreted as evidence of an epithelial malignancy. ES are known to express keratin in approximately 20% of cases and expression can be diffuse [22,23]. DSRCT characteristically expresses keratin, desmin and WT-1 (antibody against c-terminus). In addition, DSRCT can occasionally express synaptophysin [24,25] and rarely chromogranin [25,26].

As discussed, analysis for EWSR1 rearrangement and fusions can be diagnostically helpful in the appropriate setting. As a word of caution, ESWR1 gene fusions are characteristically seen in a large number of mesenchymal tumors (e.g., ES, DSRCT, clear cell sarcoma, angiomatoid fibrous histiocytoma) and rare non-mesenchymal tumors (e.g., hyalinizing clear cell carcinoma of the salivary gland). Further, in a landmark study of 102 cases of pancreatic neuroendocrine neoplasms subjected to whole-genome sequencing, EWSR1 gene fusions were seen in a small percentage (3%) [27]; these included novel fusions such as BEND2 as well as one case of EWSR1 exon 7–FLI1 exon 6 gene fusion.

A delayed sarcoma diagnosis, however, can make a difference, survival-wise. ES is particularly sensitive to appropriate chemoradiotherapy with 5-year survival rates close to 40% (data accessed on May 2020 from www.seer.gov), while high-grade NECs have a median OS of less than 1 year in the same dataset [28]. Localized cases of DSCRT and ES are still potentially curable with early identification and treatment [6,29,30]; moreover, tolerance to the ES chemotherapy is poorer after NEC-specific (platinum) treatment. Misidentification of histology can also deprive patients of clinical trial options and multidisciplinary sarcoma care, as treatments and clinical trials for patients with high-grade neuroendocrine tumors are very sparse. Ideally, every case with an ambiguous diagnosis should be sent for molecular analysis and be evaluated in a tertiary center; round cell sarcoma should be considered in tumors with a round cell pattern (alongside more common neoplasms such as lymphoma/leukemia and NECs).

Our analysis has the expected limitations associated with retrospective studies and is hampered by small numbers of misdiagnosed patients, which make it difficult to perform reliable statistical analyses. These numbers are produced from a high-volume tertiary center (115 cases in 17 years) and are likely to be similarly low, should this study be repeated in another institution. Some patients were seen only as a second opinion and were treated elsewhere with limited follow-up. Review of pathology specimens was unavailable on occasion and we speculated based on the histological reports. A follow-up analysis of misdiagnosis rates is planned for the years 2020–2025.

## 5. Conclusions

In conclusion, we have shown that patients with ES and DSCRT can frequently be misdiagnosed and this may have an effect on their survivals. Our study aims to highlight a significant problem in a vulnerable population which can benefit from timely diagnosis and appropriate, tailored treatment. We also offer some solutions based on the most common mistakes made both in the community setting and in academia.

## Figures and Tables

**Table 1:** Characteristics of patients with revised diagnoses.

ID	Sex (M/F)	Year of Dx	Age at Diagnosis	Initial Biopsy Site	Primary Tumor Site	Initial Diagnosis	Final Diagnosis	Initial Treatment	Status as of Last Contact (Alive/Dead)
1	M	2013	63	Neck	Mediastinum	Undifferentiated cancer, then PNET/lung NEC (small cell)	ELS	VAC and radiation	Alive
2	F	2013	54	Lung	Thigh	NHL (T-cell)	ELS	VAC and radiation	Alive
3	M	2013	36	Rib	Chest wall	Poorly differentiated NEC (small cell)	ES	VAC	Alive
4	M	2016	11	Bone marrow	Femur	Poorly differentiated NEC (small cell)	ES	N/A	N/A
5	M	2017	25	Mesentery	Mesentery	Poorly differentiated NEC (small cell)	ES	Etoposide and cisplatin	Dead
6	M	2018	57	Chest wall	Chest wall	Poorly differentiated NEC (small cell)	ES	Etoposide and cisplatin, avelumab	Dead
7	M	2006	29	Peritoneum	Peritoneum	Small cell cancer	DSCRT	VAC	Dead
8	F	2008	42	Uterus	Involvement of uterus, adnexa and omentum	High-grade endometrial stromal sarcoma	DSCRT	Carboplatin and paclitaxel with megestrol acetate	Dead
9	M	2011	41	Peritoneum	Peritoneum	Poorly differentiated cancer	DSCRT	N/A	Dead
10	M	2019	60	Peritoneum	Peritoneum	Poorly differentiated NEC	DSCRT	Carboplatin and etoposide, followed by pembrolizumab	Alive
11	M	2020	32	Peritoneum	Peritoneum	Small cell cancer with possible neuroendocrine differentiation	DSCRT	Cisplatin and etoposide	Alive

ES: Ewing sarcoma, ELS: Ewing-like sarcoma, DSRCT: desmoplastic small round cell tumor, NHL: Non-Hodgkin’s lymphoma, NEC: Neuroendocrine carcinoma, NOS: Not otherwise specified, PNET: Primary neuroectodermal tumor, VAC: Vincristine, Doxorubicin and Cyclophosphamide.

**Table 2:** Histological characteristics of revised patients with potential misinterpretation patterns.

ID	Molecular Diagnostics	Panel	Misdiagnosis at Sarcoma/Tertiary Care Center?	Diagnostic Challenge
1	FISH negative for *EWSR1* rearrangement	Abbott/Washington University	No	Expression of CAM5.2 and synaptophysin
2	Not done	N/A	Yes	Numerous admixed lymphocytes
3	FISH positive for *EWSR1* rearrangement	Washington University	No	Expression of CD56 and synaptophysin
4	Initial FISH negative, subsequent FISH positive for *EWSR1* rearrangement	N/A	Yes	Molecular testing initially negative, and expression of synaptophysin and pankeratin (diffuse)
5	FISH positive for *EWSR1* rearrangement, *EWSR1-FLI1* fusion reportedly also detected	Integrated Oncology Laboratories	Yes	Mesenteric origin, expression of CD56, chromogranin and synaptophysin
6	Targeted NGS panel positive for *EWSR1-FLI1* fusion	FoundationOne NGS	Yes	Expression of CD56 and synaptophysin
7	RT-PCR negative for *EWSR1-WT1* fusion	N/A	No	Expression of CD56 and chromogranin
8	nuc ish 22q12(EWSRIx3)(5′EWSRI sep 3′EWSRIx1)[14/110]/ 22q12(EWSRIx2)(5′EWSRI sep 3′EWSRIx1)[55/110]/ 22q12(EWSRIx3)[6/110]/22q12(EWSRIx2)[25/110] nuc ish 11p13(WTIx2),22q12(EWSx3),(WTI con EWSx1)[12/50]/ 11p13(WTIx2),22q12(EWSx2),(WTI con EWSx1)[11/50]/ 11p13(WTIx2),22q12(EWSx2)[21/50]	Vysis, Inc./Washington University	No	Origin in uterus
9	Negative WT1/nuc ish(EWSR1x3)(5′EWSR1 sep 3′EWSR1x2)[19/200]/(EWSR1x3)(5′EWSR1 sep 3′EWSR1x1)[10/200]/ (EWSR1x2)(5′EWSR1 sep 3′EWSR1x1)[123/200]/(EWSR1x2)[29/200]	Abbott/Washington University	Yes	Keratin expression
10	EWSR1 EWSR1(NM_005243)-WT1(NM_000378) fusion (E9;W7)	FoundationOne NGS and Washington University cytogenetics	Yes	Expression of CD56, chromogranin and synaptophysin
11	nuc ish (5′EWSR1x2-3, 3′EWSR1x2-3) (5′EWSR1 con 3′EWSR1x1) [97/100]	EWSR1 probe/Mayo clinic laboratories	No	Expression of CD56, chromogranin and synaptophysin

EWSR1: Ewing sarcoma breakpoint region 1, N/A: Not applicable, NOS: Not otherwise specified, NGS: Next-generation sequencing, FISH: Fluorescence in situ hybridization, RT-PCR: reverse transcription-polymerase reaction.

## Data Availability

Not applicable.
